# The presence of old pulmonary tuberculosis is an independent prognostic factor for squamous cell lung cancer survival

**DOI:** 10.1186/1749-8090-8-123

**Published:** 2013-05-06

**Authors:** Yiming Zhou, Zhenling Cui, Xiao Zhou, Chang Chen, Sen Jiang, Zhongyi Hu, Gening Jiang

**Affiliations:** 1 Department of Thoracic Surgery, Shanghai Pulmonary Hospital, Tongji University School of Medicine, 507 Zhengmin Rd, Shanghai, 200433, China; 2 Shanghai Key Laboratory of Tuberculosis, Shanghai Pulmonary Hospital, Tongji University, 507 Zhengmin Rd, Shanghai, 200433, China; 3 Department of Radiology, Shanghai Pulmonary Hospital, Tongji University School of Medicine, 507 Zhengmin Rd, Shanghai, 200433, China

## Abstract

**Background:**

Pulmonary tuberculosis (TB) is associated with an increased risk of lung cancer. Our study investigated whether the coexistence of an old pulmonary TB lesion is an independent prognostic factor for lung cancer survival in Chinese non-small cell lung cancer patients.

**Methods:**

We performed a retrospective review of 782 non-small cell lung cancer patients who underwent surgical resection as their primary treatment in 2006 and were followed for 5 years. The associations between lung cancer survival and the presence of old pulmonary TB lesions were assessed using Cox’s proportional hazard regression analysis adjusted for WHO performance status (PS), age, sex, smoking-status, tumor stage, and surgical approach.

**Results:**

Sixty-four of the patients had old pulmonary TB lesions. The median survival of squamous cell carcinoma patients with TB was significantly shorter than that of patients without TB (1.7 vs. 3.4 years, p < 0.01). The presence of an old pulmonary TB lesion is an independent predictor of poor survival with a hazard ratio (HR) of 1.72 (95% CI, 1.12–2.64) in the subgroup of squamous cell carcinoma patients studied.

**Conclusion:**

The presence of an old pulmonary TB lesion may be an important prognostic factor for predicting the survival of squamous cell carcinoma patients.

## Background

Non-small cell lung cancer (NSCLC) remains the leading cause of cancer death worldwide [1]. Highly effective treatments have been available for decades, but tuberculosis (TB) remains a major global health problem. In 2010, there were 8.8 million (range, 8.5-9.2 million) cases of TB and 1.1 million (range, 0.9–1.2 million) deaths from TB among the HIV-negative population [2]. Both TB and lung cancer have a large public health impact. The potential relationship between pulmonary TB and lung cancer has been a topic of active interest for several decades. At present, it is clear that pulmonary TB increases the risk of lung cancer [3-6], but not all studies agree on its utility as a prognostic factor. Recently, a study indicated that a history of pulmonary TB may be a negative prognostic factor for lung cancer survival in Caucasian patients [7], but Kuo and coworkers [8] suggested that concomitant active prolongs survival in NSCLC. Because TB is associated with lung cancer, a different question should be asked: does TB affect some types of lung cancer but not others? Luo [9] suggested that patients with pulmonary adenocarcinoma who had scar cancer or had old TB lesions had a higher probability of having EGFR mutations, especially exon 19 deletions. Moreover, squamous cell carcinoma (SCC) of the lung was found in mice subjected to chronic infection with mycobacterial tuberculosis [10]. However, an epidemiological study in a large group of TB patients and subjects without TB demonstrated that TB patients had an increased relative risk of developing adenocarcinoma but not SCC [11]. Therefore, the association between these two diseases warrants further investigation.

## Methods

We performed a retrospective review of NSCLC patients in the Department of Thoracic Surgery at Shanghai Pulmonary Hospital during a 1-year period in 2006 and followed their clinical outcomes for 5 years after surgery. This review was approved by the institutional review board of our hospital. Seven hundred eighty-two NSCLC patients who underwent surgical resection as the primary treatment were enrolled. All 782 patients received complete evaluations before surgery, including physical examination, biochemistry testing, chest computed tomography (CT) scans, radionuclide bone scans, abdominal ultrasonography, brain MRI, and bronchoscopy. The presence of old pulmonary TB lesions was revealed on the chest CT scans. The chest CT scans were evaluated for fibronodular lesions, calcified nodules, fibrotic changes, pleural thickening, and scars. All CT scans were analyzed by three independent reviewers. Lesions with calcified granulomas, fibronodular lesions, or pleural thickening suggesting previous granulomatous inflammation were regarded as old TB lesions [12]. After reviewing the chest CT scans, the three reviewers reached a final diagnosis by consensus. The TB lesions were either in the same lobe or in different lobes as the primary lung cancer lesions. The main surgical approaches used in our hospital are lobectomy, pneumonectomy, and lymph node dissection. Adjuvant therapies such as chemotherapy or radiotherapy were scheduled in our hospital during the period of this study. Complete follow-up information was obtained in 727 cases (93%); 55 cases (7%) were unable to be followed.

The *χ*^2^ test was used for categorical variables. The survival time was defined as the interval between the date of operation and the date of death or the 5-year endpoint, whichever came first. The survival curve was calculated by the Kaplan-Meier method. Univariate and multivariate analyses were performed by the Cox proportional hazards model using SPSS software (version 16.0; SPSS, Chicago, IL, USA). Clinical pathological factors, such as age, sex, WHO performance status (PS), surgical approach, smoking status, TNM stage, histologic type, and the presence of TB lesions were included in the analyses. A result was considered to be statistically significant if the p-value was less than 0.05.

## Results

One-year, 3-year, and 5-year survival rates after initial surgical resection were 75%, 53%, and 33%, respectively. Recurrent NSCLC developed in 422 patients (54%). At the time of follow-up, 523 patients had died because of local recurrence or distant metastasis (n = 396, 75.7%) or due to other causes (n =127, 24.3%). Active TB did not develop in any of the patients.

Table 1 depicts the clinical pathological characteristics of the 782 patients. Of these patients, 64 had an old pulmonary tuberculosis lesion. Only 2 patients had a clinical history of pulmonary TB and had received anti-TB treatment previously. Acid-fast smears from sputum, bronchial wash, or broncho-alveolar lavage fluid were negative in all NSCLC patients. The mean age at diagnosis of lung cancer was 60 years. Approximately two-thirds of the 782 subjects in this study were smokers or had a history of smoking at the time of diagnosis of lung cancer. In the TB group, squamous cell carcinoma was the most common histological type (51.6%), followed by adenocarcinoma (43.8%). In the non-TB group, adenocarcinoma was the most common histological type (52.6%), followed by squamous cell carcinoma (37.5%).

**Table 1:** Patient characteristics

	**All NSCLC**				**SCC**			
***Characteristic***	**Number (% of total)**	**Without tuber-culosis (%)**	**With tuber-culosis (%)**	**p-value**	**Number (% of SCC)**	**Without tuber-culosis (%)**	**With tuber-culosis (%)**	**p-value**
***Number of subjects***	782(100)	718(91.8)	64(8.2)		302(100)	269(89.1)	33(10.9)	
***Age at diagnosis****(years) (mean±SD)*	60±10.2	60±10.2	61±9.8	0.43	62±9.3	62±9.4	63±7.9	0.83
***PS***				1.0				0.39
***0***	98	90	8		35	33	2	
***1***	624	628	56		267	236	21	
***Sex***				0.06				0.13
*Male*	600(76.7)	545(75.9)	55(85.9)		285(94.4)	252(93.7)	33(100)	
*Female*	182(23.3)	173(24.1)	9(14.1)		17(5.6)	17(6.3)	0(0)	
***Smoking status***				0.18				0.70
*Never*	240(30.7)	225(31.3)	15(23.4)		48(15.9)	42(15.6)	6(18.2)	
*Smoker or ex-smoker*	542(69.3)	493(68.7)	49(76.6)		254(84.1)	227(84.4)	27(81.8)	
***Stage of lung cancer at diagnosis***^***c***^				0.18				0.16
*I*	315(40.3)	287(40.0)	28(43.8)		123(40.7)	114(42.4)	9(27.3)	
*II*	157(20.1)	141(19.6)	16(25.0)		60(19.9)	50(18.6)	10(30.3)	
*III*	246(31.5)	227(30.6)	19(29.7)		110(36.4)	96(35.7)	14(42.4)	
*IV*	64(8.2)	63(8.8)	1(1.6)		9(3.0)	9(3.3)	0(0.0)	
***Histologic type***				0.06				
*SCC*	302(38.6)	269(37.5)	33(51.6)					
*Adenocarcinoma*	406(51.9)	378(52.6)	28(43.8)					
*Other*^*a*^	74(9.5)	71(9.9)	3(4.7)					
***Surgical approach***				0.10				0.07
*Lobectomy*	685(87.6)	633(88.2)	52(81.2)		245(81.1)	222(82.5)	23(69.7)	
*Pneumonectomy*	97(12.4)	85(11.8)	12(18.8)		57(18.9)	47(17.5)	10(30.3)	
***Median survival****(year)*	3.4	3.4	3.0	0.50	3.2	3.4	1.7	<0.01^b^

^a^ Including adenosquamous carcinoma, sarcomatoid carcinoma, and undifferentiated carcinoma.

^b^ A p-value less than 0.05 was considered statistically significant.

^C^ All patients were re-staged according to the 7^th^ edition lung cancer staging. SCC = squamous cell carcinoma.

Table 2 shows the adjuvant treatment after surgery. Notably, some patients declined the adjuvant therapy due to personal reasons, which were distributed randomly.

**Table 2:** **Adjuvant therapy**^**b **^**after surgery for squamous cell carcinoma**

**Stage of lung cancer at diagnosis**^**a**^	**Without TB(%)**	**With TB(%)**	**p-value**
I	114	9	0.91
Without adjuvant therapy	40(35.1)	3(33.3)	
With adjuvant therapy	74(64.9)	6(66.7)	
II	50	10	0.78
Without adjuvant therapy	12(24.0)	2(20)	
With adjuvant therapy	38(76.0)	8(80)	
III &IV	105	14	0.96
Without adjuvant therapy	23(21.9)	3(21.4)	
With adjuvant therapy	82(78.1)	11(78.6)	

^a^All patients were re-staged according to the 7th edition of the TNM-staging of lung cancer.

^b^Adjuvant therapy included chemotherapy and radiotherapy. We arranged these treatments according to the NCCN guidelines.

**Figure 1:**
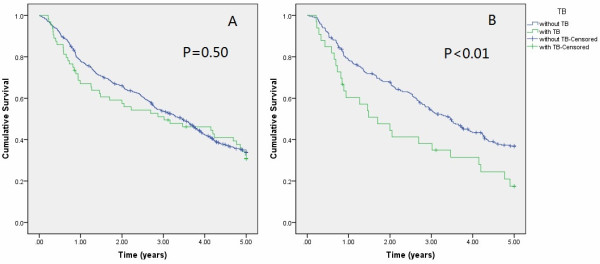
**Kaplan-Meier survival curve.** (**A**) All NSCLC patients with and without active TB (log rank test, P = 0.50). Green line: patients with TB. Blue line: patients without TB. (**B**) SCC patients with and without active TB (log rank test, P < 0.01). Green line: patients with TB. Blue line: patients without TB.

The comparison of lung cancer survival outcomes in patients with and without TB is shown in Figure 1A (all NSCLC patients) and Figure 1B (SCC patients). Among the entire NSCLC group, there was no significant difference in survival between patients with and without TB. However, the SCC patients with TB lesions had a significantly shorter median survival time than the SCC patients without TB lesions (1.7 vs. 3.4 years, p < 0.05). In patients with adenocarcinoma and other histologic subtypes, survival did not significantly differ between patients with and without tuberculosis.

Table 3 shows the crude and adjusted hazard ratios (HRs) for survival time in SCC patients. The types of surgical approach, sex, stage of cancer, smoking status, PS, and the presence or absence of TB lesions were associated with survival in univariate and multivariate analysis. The crude HR for the presence of old TB lesions showed a 1.73-fold increased risk (95% CI: 1.14–2.63), and the adjusted HR of the presence of old TB lesions showed a 1.72-fold increased risk (95% CI: 1.12–2.64).

**Table 3:** Prognostic factors for SCC survival outcome as determined by univariate and multivariate analyses

**Variables**	**Univariate**		**Multivariate**	
	**HR (95% CI)**	**p-value**	**HR (95% CI)**	**p-value**
**TB**				
Without TB	1.00	<0.01^a^	1.00	<0.05^a^
With TB	1.73(1.14~2.63)		1.72 (1.12~2.64)	
**Sex**				
Female	1.00	0.95	1.00	0.5
Male	0.98(0.54~1.76)		0.84(0.45~1.57)	
**Stage of lung cancer**^**b**^				
I	1.00		1.00	
II	2.08(1.35~3.20)	<0.01^a^	1.89(1.12~2.93)	<0.01^a^
III	6.26(4.33~9.05)	<0.01^a^	5.27(3.58~7.74)	<0.01^a^
IV	7.84(3.62~17.05)	<0.01^a^	6.56(2.99~14.35)	<0.01^a^
**Smoking status**				
Non-smoker	1.00	0.44	1.00	0.92
Smoker or ex-smoker	0.86(0.59~1.25)		1.01(0.69~1.50)	
**Surgical approach**				
Lobectomy	1.00	<0.05^a^	1.00	0.50
Pneumonectomy	1.50(1.06~2.13)		0.88(0.61~1.27)	
**PS**				
0	1.00	<0.01^a^	1.00	<0.05^a^
1	8.21(3.33~20.00)		4.81(1.94~11.90)	

^a^A p-value less than 0.05 was considered significant. HR = hazard ratio.

^b^All patients were re-staged according to the 7th edition lung cancer staging. SCC = squamous cell carcinoma. TB = tuberculosis.

## Discussion

To our knowledge, this is the first study to demonstrate the presence of an old pulmonary TB lesion as an independent prognostic risk factor for squamous cell lung cancer survival. In 1996, it was reported that Taiwanese lung cancer patients who present initially with active TB do not survive as long as those without active TB [13]. The patients included in the 1996 study were defined as TB-positive only if they were diagnosed with TB up to 2 years before or after their diagnosis of lung cancer, and the survival analysis was confounded by an important prognostic factor of lung cancer, i.e., TNM stage. These constraints may have introduced selection bias as well as information bias. Recently, Heuvers et al. reported that a history of pulmonary TB may be an important prognostic factor for the survival of lung cancer [7]. However, their study was limited by recall bias, as not all subjects may have remembered their respiratory TB or may have been unaware of TB infection in the past. Moreover, this study did not include the WHO PS of the lung cancer cases, and the authors were not aware of the treatment of the patients. Kuo [8] demonstrated that concomitant active TB in NSCLC was associated with improved survival. However, all 276 patients included in Kuo’s study were in advanced stages of NSCLC (stage III and stage IV). Their samples are therefore not representative of the general lung cancer population. There are two major prognostic factors for lung cancer: WHO PS and disease stage. In our study, the patients who underwent surgical resection were typically at a PS level of 0–1. In contrast to these previous studies, the larger number and more representative nature of the samples collected in the present study provide more reliable statistical power for assessing the relationship between lung cancer and TB.

Although TB did not affect overall survival in the whole NSCLC population in our study, Luo [9] recently suggested that patients with pulmonary adenocarcinoma and TB lesions had a higher probability of having EGFR mutations. Exon 19 deletions occurred more frequently in patients with old TB lesions than in patients without TB lesions. Those patients with old TB lesions who had EGFR mutations or exon 19 mutations survived longer than those who did not. Thus, we divided all 782 cases into 3 subgroups according to their histologic type. Although TB may affect the immune system in all NSCLC cases, a correlation between the presence of old TB lesions and survival outcomes was observed only in the SCC subgroup. In the patients with EGFR-activating mutations, EGFR-TKI treatment can be continued. This treatment is effective against EGFR-activated cancers, providing an advantage in cancer control of this group. In our study, TB did not impact the survival of adenocarcinoma patients. Our study is limited by the lack of information regarding the EGFR mutation status in our patients; however, most cancer-associated EGFR mutations occur in adenocarcinomas of the lungs. Therefore, the shorter survival observed in the SCC patients with old TB lesions is not likely to be due to EGFR mutations.

Similar to other clinical studies with larger sample sizes, in our study, SCC was the most common histological type in the TB group [8,13,14]. Nalbandian [10] presented experimental evidence that chronic TB infection can trigger a series of events that leads to extensive remodeling of lung tissue or activation of a typical differentiation pathway resulting in malignant transformation such as squamous cell metaplasia. These authors also observed the genetic control of SCC development induced by TB infection.

## Conclusions

In conclusion, the presence of pulmonary TB is a negative prognostic factor for SCC of the lung in Chinese patients. More research is necessary to define common immunological pathways in TB and lung cancer.

## Abbreviations

TB: Tuberculosis; NSCLC: Non-small cell lung cancer; SCC: Squamous cell carcinoma; PS: Performance status; EGFR: Epidermal growth factor receptor.

## Competing interests

The authors declare that they have no competing interests.

## Authors’ contributions

YMZ, GNJ, and ZYH participated in the design of the study and performed the statistical analysis. CC, XZ, SJ, and ZLC conceived of the study, participated in its design and coordination, and helped draft the manuscript. All authors read and approved the final manuscript.
